# Electrochemical Metal Recycling: Recovery of Palladium from Solution and In Situ Fabrication of Palladium-Carbon Catalysts via Impact Electrochemistry

**DOI:** 10.1021/jacs.2c08239

**Published:** 2022-09-30

**Authors:** Abiola V. Oladeji, James M. Courtney, Marcos Fernandez-Villamarin, Neil V. Rees

**Affiliations:** School of Chemical Engineering, University of Birmingham, Edgbaston, Birmingham B15 2TT, U. K.

## Abstract

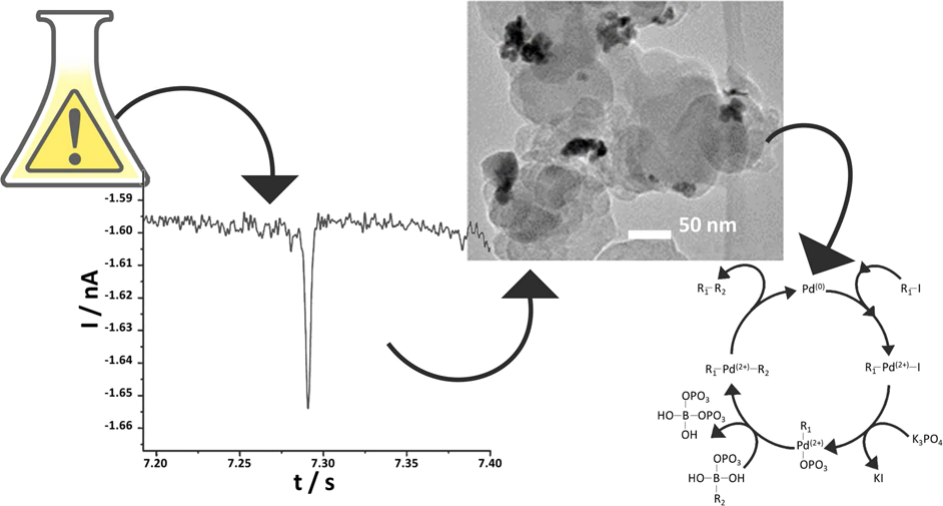

Recycling of critical materials, regeneration of waste, and responsible catalyst manufacture have been repeatedly documented as essential for a sustainable future with respect to the environment and energy production. Electrochemical methods have become increasingly recognized as capable of achieving these goals, and “impact” electrochemistry, with the advantages associated with dynamic nanoelectrodes, has recently emerged as a prime candidate for the recovery of metals from solution. In this report, the nanoimpact technique is used to generate carbon-supported palladium catalysts from low-concentration palladium(II) chloride solutions (i.e., a waste stream mimic) as a proof of concept. Subsequently, the catalytic properties of this material in both synthesis (Suzuki coupling reaction) and electrocatalysis (hydrogen evolution) are demonstrated. Transient reductive impact signals are shown and analyzed at potentials negative of +0.4 V (vs SCE) corresponding to the onset of palladium deposition in traditional voltammetry. Direct evidence of Pd modification was obtained through characterization by environmental scanning electron microscopy/energy-dispersive X-ray spectroscopy, inductively coupled plasma mass spectrometry, X-ray photoelectron spectroscopy, transmission electron microscopy, and thermogravimetric analysis of impacted particles. This showed the formation of deposits of Pd0 partially covering the 50 nm carbon black particles with approximately 14% Pd (wt %) under the conditions used. This material was then used to demonstrate the conversion of iodobenzene into its biphenyl product (confirmed through nuclear magnetic resonance) and the successful production of hydrogen as an electrocatalyst under acidic conditions (under cyclic voltammetry).

## 1 Introduction

Platinum group metals (PGMs) are significant critical metals because of their wide range of applications, most notably as catalysts.^1−3^ Palladium is a common catalyst, utilized in many applications and processes such as environmentally important automotive catalytic converters, future energy use in hydrogen storage and production, and large-scale chemical synthesis.^4−15^ The Suzuki coupling reaction for example is an industrially important reaction frequently used in pharmaceutical drug synthesis, the formulation of agrochemicals, and polymer production.^16−28^

To improve both performance and economic use, PGM catalysts are often used as supported nanoparticles (NPs), where the support particles disperse the catalytic material to a greater extent, increasing exposure of the catalytic surface area. Carbon materials such as carbon black (CB), graphite, and graphene have been extensively studied as supports,^6,29−37^ as they are inexpensive and possess useful properties such as a high electrical conductivity and large surface areas while being mechanically and chemically stable.^38−45^

Pd-modified carbon nanostructured catalysts are extensively used in a range of applications: from electrochemical sensors and organic synthesis to the oxidation of formic acid and reduction of oxygen in fuel cells.^46−59^ However, the use of Pd can lead to environmental pollution via release of contaminated waste solutions and particles.^60−64^ For example, the World Health Organization has reported concentrations of 260 μg kg^–1^ in sewage sludge and up to 4.7 mg kg^–1^ in waste discharged from the jewelry industries.^65^ Other sources of soluble palladium (often PdCl_2_) such as e-waste (electroplating and printed circuit boards) can result in concentrations of 1500 mg L^–1^ in waste streams.^66^ Concentrations in natural waters are significantly lower, with Pd concentrations of 22 ng L ^–1^ and 70 pg L^–1^ being detected in fresh water and sea water, respectively.^67^ Palladium metal has historically been considered relatively low in toxicity; however, its compounds such as palladium(II) chloride are highly toxic and carcinogenic to wildlife even in minute amounts. For example, a minimum 24 h lethal concentration of 7 mg has been reported for the freshwater fish *medaka,* and LD_50_ values in rats reach between 0.02 and 1.13 mmol kg^–1^ bodyweight;^67,68^ hence, it is important to maximize recovery and clean waste already existing for both environmental and economic reasons.^4,67,69−71^

The primary method used to recover spent Pd catalysts is hydrometallurgy which is limited because of the significant volumes of toxic and expensive reagents needed and the further production of hazardous waste such as nitric oxides.^4,72−74^ These methods, often requiring the pretreatment of Pd, have been able to achieve recovery within the range of 58–97%^75−83^ but are considered ineffective for the removal of metals from waste solutions at low concentrations resulting in waste storage challenges and reduced profit.^66,84,85^ Therefore, more environmentally sustainable methods of Pd nanoparticle synthesis and recovery have been investigated.^37,66,86,87^ Electrodeposition is considered a practical method for metal recovery because of its operational feasibility, with the deposition controlled through the potential applied and metal ion concentration,^88−91^ and traditional electrodeposition methods have many industrial applications.^92−98^ The use of electrochemical systems such as galvanic reduction and recovery from ionic liquids after solvent extraction have been explored within the literature as a method of Pd extraction achieving a recovery of 90–99%.^66,99^ Higher recoveries were observed in systems increasing mass transport via the use of rotating electrodes or flow of the plating solution.^100,101^ However, overall recovery or recycling efficiency is not necessarily easily compared because studies often focus on one part of the recycling process (usually recovery, rather than separation, dissolution, etc.).

In this work, we consider the recovery process from solution: particle impact electrochemistry has been investigated as a method for in situ synthesis of Pd-modified carbon black nanoparticles (Pd/CB NPs) from the recovery of palladium from solutions containing low concentrations of PdCl_2_ as a proxy for industrial waste streams.

The impact electrochemistry technique involves nanoparticles suspended in solution moving under Brownian motion and occasionally “colliding” with a substrate electrode held at a suitable potential.^102−105^ While the particle-electrode interaction is usually referred to in terms of a “collision”, there is of course no requirement for physical contact: provided the particle approaches sufficiently close to reach the plane of electron transfer (i.e., electron tunneling distance), then electroreduction or oxidation can occur. Upon collision, the nanoparticles provide a surface at which solution species can be oxidized or reduced,^106−123^ resulting in transient current signals that can be analyzed to determine factors such as particle size, concentrations, and kinetics.^124−129^ The shape of the transient signal can also be used to infer the type of collision (or interaction) between the particle and electrode, ranging from apparently fully elastic (rebound) impacts to fully inelastic (“hit and stick”) impacts which appear to depend on a range of factors such as electrode and particle materials, surface groups, solution conditions, and so on. The extent of the electrochemical reaction occurring on the surface of the particle during a collision is therefore dependent on the conditions of the impact, most notably the electrochemical kinetics of the reaction and the duration of the particle-electrode “contact”. Once the particle moves away from the plane of electron transfer, no further (heterogeneous) electron transfer should occur. In some cases, the particles and substrate electrodes are chosen to be different materials: if the desired electrochemical reaction has significantly faster kinetics on the particle compared to the substrate, it is possible to only observe reactions at the particles. Where there is little or no difference in kinetics, the electrochemical reaction occurs at both the substrate and particles, which often makes observing impact signals challenging because of the respective current magnitudes.

Impact electrochemistry has previously been reported as a method of reducing metallic ions onto metallic nanoparticles (or cores) via both bulk and underpotential deposition processes.^130−132^ However, the deposition onto nonmetallic cores (for metal recovery) via impact electrochemistry has only recently been described, for the deposition of copper ions onto fly-ash cenosphere particles.^133^ The use of nonmetallic materials potentially increases the economic viability, flexibility, and sustainability of this technique as it reduces reliance on expensive and potentially critical core materials.

In this paper, we report the first metal on carbon deposition by impact electrochemistry for the case of Pd on CB, using the method to fabricate Pd/CB nanoparticles which were then characterized and directly used, as a proof of concept, to catalyze the hydrogen evolution and Suzuki coupling reactions. Under nonoptimized conditions, the fabrication process recovered >85% of Pd from solution in 26 h, suggesting the viability for this technique in recovery/recycling of metals.

## 2 Experimental Section

All chemicals used were obtained commercially and used without further purification, namely, palladium chloride (99.99%, Sigma-Aldrich), potassium chloride (99.0–100.5%, Alfa Aesar), potassium sulfate (99.0%, Alfa Aesar), sulfuric acid (95.0–98.0%, Sigma-Aldrich), hydrochloric acid (37.0%, Honeywell), and 50 nm-diameter CB nanoparticles (Fuel cell store). All solutions were made using ultrapure water of resistivity ≥18.2 MΩ cm (MilliQ, Millipore).

### 2.1 Electrochemistry

Electrochemical experiments were performed using a three-electrode cell in a Faraday cage. The working electrodes used were a glassy carbon macroelectrode (GC, 3 mm diameter, BASi Inc) and carbon fiber (CF) microelectrodes of diameters 33 μm (ALS Inc.) and 9 μm (made in-house using pitch-derived CF from Goodfellow Cambridge Ltd). All working electrodes were thoroughly polished with 3 μm diamond paste and alumina suspensions of 1, 0.3, and 0.05 μm sequentially, on a microcloth pad (all from Buehler Inc., USA). A saturated calomel electrode (SCE, ALS Inc) was used as a reference electrode and a graphite rod (3 mm diameter, Goodfellow Cambridge Ltd.) as the counter electrode. For impact studies, the SCE reference electrode was placed in a separate fritted compartment to prevent cross-contamination. Unless otherwise stated, a solution containing 0.01 M potassium chloride, 0.01 M hydrochloric acid, and 0.5 mM palladium(II) chloride was used. In this solution, the PdCl_3_^–^ ion is more prevalent than PdCl_4_^2–^ because of the relatively low chloride concentration (see the Supporting Information for calculation). A bulk solution of CB NPs was prepared by adding CB NPs to ultrapure water and sonicating for 1 h before use. The desired CB NP concentration of 50 pM was prepared using aliquots of the CB NP bulk solution. Solutions were thoroughly degassed using nitrogen gas (oxygen-free, BOC Gases plc), and a nitrogen atmosphere was maintained throughout the experiments. For impact experiments, the solution was bubbled with nitrogen regularly to agitate the particle suspension and inhibit aggregation. For particle-modified GC voltammetry, the GC electrode was prepared via a drop-cast method where 5 μL of the relevant NP suspension was added to a polished GC electrode surface and allowed to dry under nitrogen.

Standard electrochemical measurements were conducted using an Autolab 128 N (Metrohm-Autolab BV, Netherlands) potentiostat controlled via a PC running NOVA 2.1 software conducting both linear sweep voltammetry and chronoamperometry scans. Particle impact chronoamperometric scans were performed using a bespoke low noise potentiostat,^133^ with a sampling rate of 100 kHz. All data were processed using a combination of Microsoft Excel and Origin Pro 2021. Unless stated otherwise, impact electrochemical data were analyzed following electronic filtration (digital) at 250 Hz in order to improve the signal-to-noise ratio and facilitate analysis. The design of the potentiostat is such that charge is conserved by the filter, as shown elsewhere.^133−135^

For the bulk synthesis of Pd-modified carbon nanoparticles (Pd/CB) using impact electrochemistry, a polished graphite plate working electrode (6.25 cm^2^) with a larger area graphite counter electrode (Alfa Aesar) and a SCE reference electrode were used. Long-term chronoamperography (potential held at −0.1 V) was conducted in 500 mL of palladium solution with 20 nM 50 nm CB NPs to produce Pd/CB NP samples that had been modified for 168 h for imaging and testing for catalytic activity. The reacted Pd/CB samples were filtered using 0.02 μm anodisc membrane filters (Cytiva) and repeatedly rinsed with ultrapure water before drying. An analogous experiment was conducted for 24 h to fabricate Pd/CB nanoparticles which were tested electrocatalytically via the hydrogen evolution reaction (HER) by drop casting 5 μL (giving a catalyst mass of 8.5 g m^–2^) onto a bare GC electrode.^133,136^ To prepare the three catalyst inks, unmodified CB NPs, Pd/CB NPs (modified via impacts), and commercial 10% Pd on CB nanoparticles (Sigma-Aldrich) were added to ultrapure water respectively. This was followed by the addition of NafionTM dispersion D1021 (Fuel Cell Store) to each catalyst ink to achieve 10% of total mass (i.e., carbon plus Pd). Cyclic voltammetry (CV) scans were then conducted at 100 mV s^–1^ in a solution of 0.01 M H_2_SO_4_ and 0.09 M K_2_SO_4_ in the potential window of 1.0 to −1.7 V vs SCE using a graphite rod as the counter electrode.

To analyze the rate of Pd recovery, experiments were conducted for 26 h, as above, with and without the addition of CB NPs where the nitrogen flow rate was kept constant at 5 L min^–1^, and 3 mL aliquots were extracted at designated time intervals. The samples were filtered and diluted with ultrapure water for inductively coupled plasma optical emission spectrometry (ICP-OES) using a PerkinElmer Optima 8000. A calibration curve was generated with a 10 ppm multielement calibration standard for ICP (Agilent), in ultrapure water at concentrations of 5–0.01 ppm. The samples were then analyzed, and the concentration of palladium was read from the calibration curve which has an upper concentration limit of 500 ppm.

### 2.2 Material Characterization of Pd-Modified CB NPs

Modified and unmodified CB nanoparticle samples were characterized using environmental scanning electron microscopy (ESEM) with energy-dispersive X-ray spectroscopy (EDX), transmission electron microscopy (TEM) with EDX, X-ray photoelectron spectroscopy (XPS) analysis, and inductively coupled plasma mass spectrometry (ICP-MS). For the ESEM/EDX characterization, 5 mg of CB nanoparticle samples were added to the surface of carbon tape (Agar Scientific) and then analyzed using a Philips XL30 FEG ESEM. For TEM, samples were drop-cast onto grids and imaged using a JEM-2100F Field Emission Electron Microscope operated at 200 kV, equipped with a Gatan Orius SC1000 CCD camera (performed at NMRC, University of Nottingham). HAADF-STEM data were acquired using JEOL DF detectors, and EDX data were acquired using an Oxford Instruments X-Max 80 EDX detector. For the XPS characterization, the samples were analyzed (at NMRC, University of Nottingham) using a Kratos Liquid Phase Photoelectron Spectrometer (LiPPS, in dry sample mode) with monochromated Al KαX-ray source (1486.6 eV). This was operated at 10 mA emission current and 12 kV anode potential (120 W). For the wide scan, a pass energy of 80 eV was used (run with a step size of 0.5 eV) while the high-resolution scan was conducted with a pass energy of 20 eV (with a step size of 0.1 eV). Data processing was conducted using CASAXPS software (version 2.3.20) with Kratos sensitivity factors (RSFs) to determine the atomic percentage values from the peaks.

For the ICP-MS analysis, three samples were dissolved in aqua regia, then diluted using ultrapure water to obtain 1% aqua regia at a concentration of 1 ppm (CB content), and filtered with 0.45 μm syringe filters (Starlab Group Ltd). The samples were then analyzed using ICP-MS (Nexion 300X ICP-MS, PerkinElmer) with a limit of detection of 10 ppt. A calibration curve was generated as described for the ICP-OES analysis ranging from 1 ppb to 1 ppm.

Thermogravimetric analysis (TGA) (sample weight 8.61 mg) was performed using NETZSCH TG 209 F1 in an aluminum oxide crucible at a heating rate of 10 °C min^–1^ from 25 to 900 °C under nitrogen purging (10 mL min^–1^), and sample weights were additionally measured using the nanobalance (Sartorius) before and after thermal treatment.

### 2.3 Catalysis of the Suzuki Coupling Reaction

For the Suzuki coupling reaction, a solution of 184 mg of phenylboronic acid (≥97.0%, Sigma-Aldrich), 426 mg of potassium phosphate tribasic (≥ 98.0%, Sigma-Aldrich), and 20 mL of ultrapure water was added to a three-necked round bottom flask and stirred (450 rpm) for 20 min. To this mixture 115 μL of iodobenzene (98.0%, Sigma-Aldrich) and *ca*. 30 mg of Pd-modified carbon nanoparticles were added while stirring. The mixture was stirred and refluxed in a silicone oil bath at 80 °C for 6 h. After cooling, the organic phase was extracted three times using 20 mL of ethyl acetate (Sigma-Aldrich) and then dried using anhydrous sodium sulfate (Sigma-Aldrich). The sodium sulfate was removed from the organic phase using 5–13 μm filter paper (Fisherbrand), and then the ethyl acetate was evaporated. The powdered sample was dissolved in CDCl_3_ for ^1^H NMR analysis using a Bruker 400 MHz NMR spectrometer where four scans were conducted with an acquisition time of 4.7 s and relaxation delay of 2 s. Data were analyzed using Mestrenova software (version 14.0.0).

## 3 Results and Discussion

### 3.1 Impact Deposition of Pd onto CB Nanoparticles

First, preliminary experiments were performed to confirm the deposition of Pd onto carbon surfaces and to determine at which potentials the deposition onto CB NPs during impacts might occur. To do this, the deposition of Pd from a solution containing 0.5 mM PdCl_2_, 0.01 M KCl, and 0.01 M HCl was investigated using macroelectrode CV at a voltage scan rate of 100 mV s^–1^ to determine the onset potential on the bare GC electrode, where onset is defined here as the potential at which the measured current density was −0.5 mA m^–2^.^137−139^

**Figure 1:**
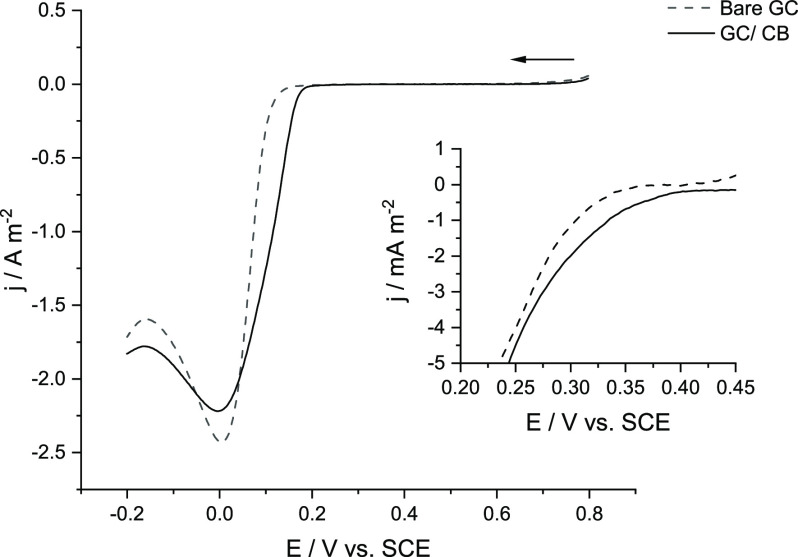
Reductive voltammetric scans of Pd deposition from a solution of 0.5 mM PdCl_2_, 0.01 M KCl, and 0.01 M HCl onto the surface of 3 mm bare GC (·····) and 50 nm CB NP-modified GC (—) electrode where the inset shows the magnified onset potentials where Pd deposition commences. The voltage scan rate was 100 mV s^–1^ for all scans.

Figure 1 shows the reductive segments of CV scans showing the deposition of palladium on the surface of 3 mm bare GC and 50 nm carbon black-modified GC electrodes. The CB-modified GC electrode was prepared via a drop-cast method where 5 μL of the CB NP suspension was added to a polished GC electrode surface and allowed to dry, resulting in a surface (particle) concentration of 66 pmol m^–2^. The deposition of palladium is known to be a two-electron transfer,^46,54^

Pd^2+^_(aq)_ + 2e^–^ → Pd_(s)_, where the nucleation mechanism and morphology of the deposited Pd are influenced by deposition conditions such as applied potential and Pd concentration.^88^ The literature records the bulk deposition process to occur at potentials lower than *ca*. +0.3 to +0.4 V (vs SCE) depending on the carbonaceous material.^88,140^ This is in good agreement with the recorded data (Figure 1) showing the onset at +0.29 V (vs SCE) for the bare GC and +0.38 V (vs SCE) on the CB NP-modified GC. The proximity of these onset potentials suggests that it is unlikely that Pd will deposit onto CB NPs during impacts without some degree of background deposition onto the substrate GC electrode. It has previously been shown that at more positive potentials (lower overpotential) the electronucleation of Pd occurs via a 3D instantaneous mechanism while more negative potentials (higher overpotential) result in a 3D progressive nucleation mechanism.^88,141,142^

Having determined the onset potentials for Pd deposition on GC and CB NPs under these conditions, chronoamperometric scans were conducted in the presence and absence of nanoparticles to investigate whether transient impact events occurred. The chronoamperometric scans were conducted using a three-electrode cell in a 0.5 mM palladium solution (as described previously) where either a 9 μm or a 33 μm diameter CF electrode acted as the substrate surface.

**Figure 2:**
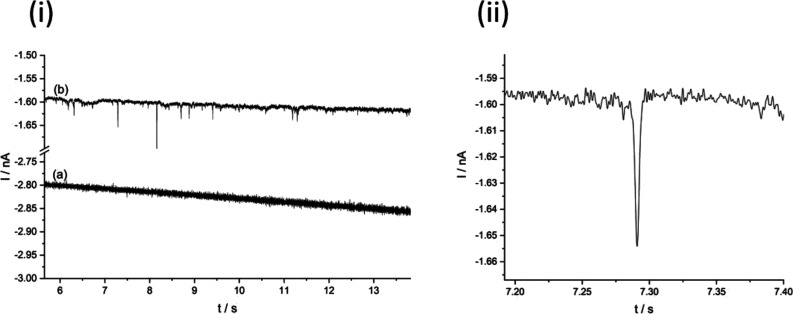
(i) Typical segments of chronoamperometric scans conducted at −0.1 V vs SCE using a 9 μm CF electrode: (a) before and (b) after the addition of 50 pM 50 nm CB nanoparticles to a solution of 0.5 mM PdCl_2_, 0.01 M KCl, and 0.01 M HCl. (ii) A magnified reductive signal from scan (b) at *t* = 7.29 s.

Figure 2 displays two chronoamperometric scans conducted using a 9 μm CF microelectrode at −0.1 V vs SCE before (a) and after (b) the addition of CB nanoparticles where scan (a) is void of reductive peaks. Upon the addition of nanoparticles, peaks were observed indicating the deposition of palladium onto the CB NPs upon impact. At this potential, Pd deposition may occur at CB NPs as well as the substrate GC electrode (see Figure 1), and so the measured currents reflect the capacitance of the GC substrate as well as any faradaic currents because of Pd deposition. The difference in currents of (a) and (b) is ascribed to fluctuations because of natural convection given the length of each chronoamperometric scan. Impact studies were conducted on both 9 and 33 μm CF electrodes: the former was used predominantly for potentials close to the onset of deposition, where transient signals were small, and optimal signal-to-noise ratios were required. Although the smaller electrode provided lower noise levels due to lower capacitance (see Supporting Information Section A), its smaller area also led to less frequent transient signals, and hence for convenience, the larger CF substrate electrodes were used at potentials further from onset to provide a greater quantity of data more rapidly.

**Figure 3:**
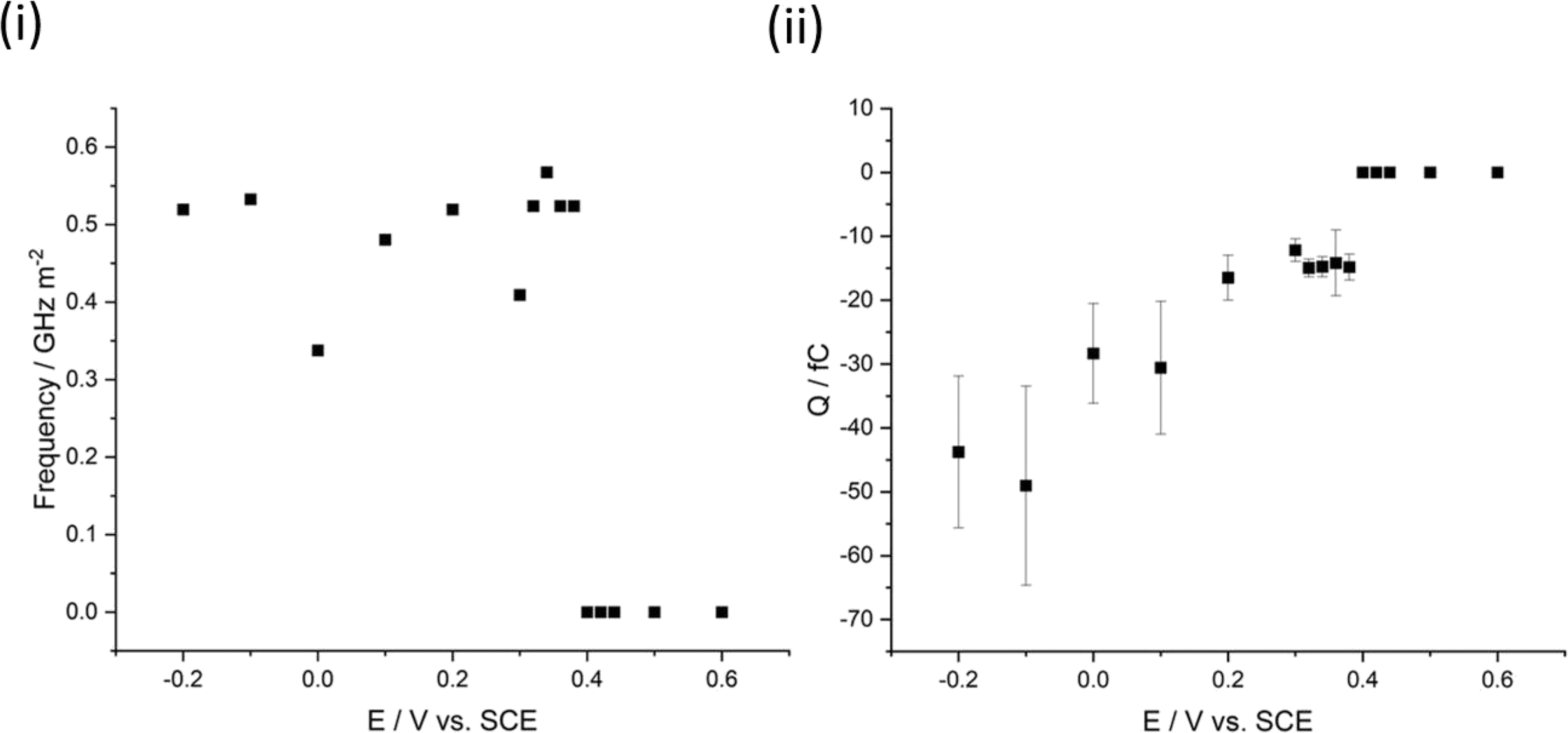
(i) shows the frequency of reductive transient peaks detected during 30 s chronoamperometric scans at different potentials ranging from +0.6 to −0.2 V vs SCE using both 33 and 9 μm CF electrodes and (ii) displays the mean calculated charge passed during transient deposition events analyzed using unfiltered peaks. All scans were conducted in a 5 mL solution of 0.5 mM PdCl_2_, 0.01 M KCl, and 0.01 M HCl.

**Figure 4:**
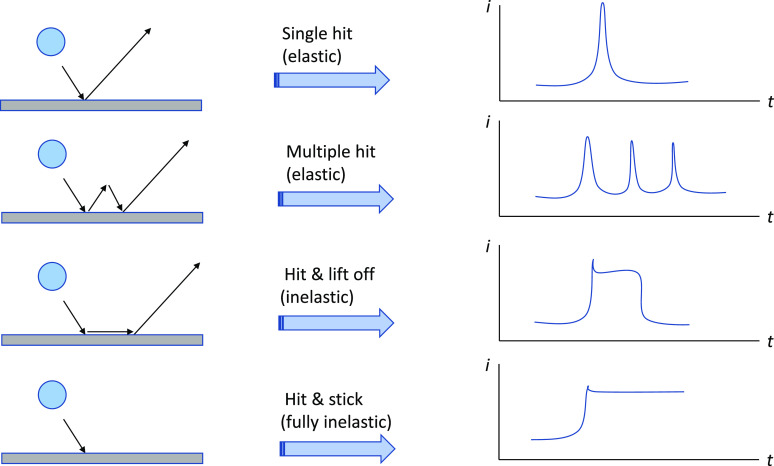
Schematic showing some possible impact scenarios and simplified transient signals.

Further impact studies were conducted by performing multiple chronoamperometric scans using 50 pM CB NPs at potentials between +0.6 and −0.2 V vs SCE. The impact frequency was determined at each potential by dividing the total number of observed impact signals by the total time of all scans, as seen in Figure 3i and was in the range of 0.34–0.57 GHz m^–2^ (within the literature a frequency range of 0.7–3.7 GHz m^–2^ is documented for metal deposition impacts using *ca*. 20 pM metallic cores.^130,133^). From Figure 3, reductive peaks can be observed at potentials negative of +0.4 V vs SCE, consistent with the onset observed in Figure 1. At all potentials, the impact signals had a similar duration, indicating that deposition of Pd occurred during approximately elastic collisions with the electrode (if deposition occurred at NPs that had preadsorbed to the GC surface, then step-like signals would be observed, see Figure 4 for a schematic). The role of preadsorbed Pd^2+^ is less straightforward to quantify and may well prove to be key to electrodeposition during impacts, especially where reduction kinetics may be less than reversible.

Integration of the CB NP reductive transients produced the charge associated with Pd deposition on individual nanoparticles (see Supporting Information Section B Table T1).^133^Figure 3ii shows the average charge of impacting CB NPs at different potentials ranging from +0.6 to −0.2 V vs SCE where at potentials more reductive than +0.4 V, charge ranging from −12.2 to −49.0 fC is observed, seemingly increasing with increased overpotential. The theoretical Pd coverage can be calculated based on the charge transferred during impacts (see Supporting Information Section B for details). For example, at +0.38 V (vs SCE) an average charge of −14.8 ± 2.1 fC corresponds to a coverage of 110 ± 15%, increasing to 365 ± 116% at a more reductive potential of −0.1 V (vs SCE) where the charge was −49.0 ± 15.6 fC.

### 3.2 Characterization of Pd-Modified CB NPs

To confirm palladium deposition on the CB nanoparticles, a 24 h impact experiment was conducted using a graphite plate electrode (area 6.25 cm^2^) held at a potential of −0.1 V (vs SCE) in 500 mL of a solution containing 0.5 mM PdCl_2_, 0.01 M KCl, and 0.01 M HCl. The upscaling of the experiment was required to produce sufficient mass of impacted CB NPs to analyze. A concentration of 20 nM CB NPs was added to the solution and agitated continually with a nitrogen gas stream for the 24 h period. An analogous experiment was conducted at +0.6 V vs SCE, where no transient impact peaks due to Pd reduction were expected. The two samples held at −0.1 V and +0.6 V were rinsed thoroughly with deionized water during filtration using 0.02 μm anodisc inorganic membrane filters before drying.

**Figure 5:**
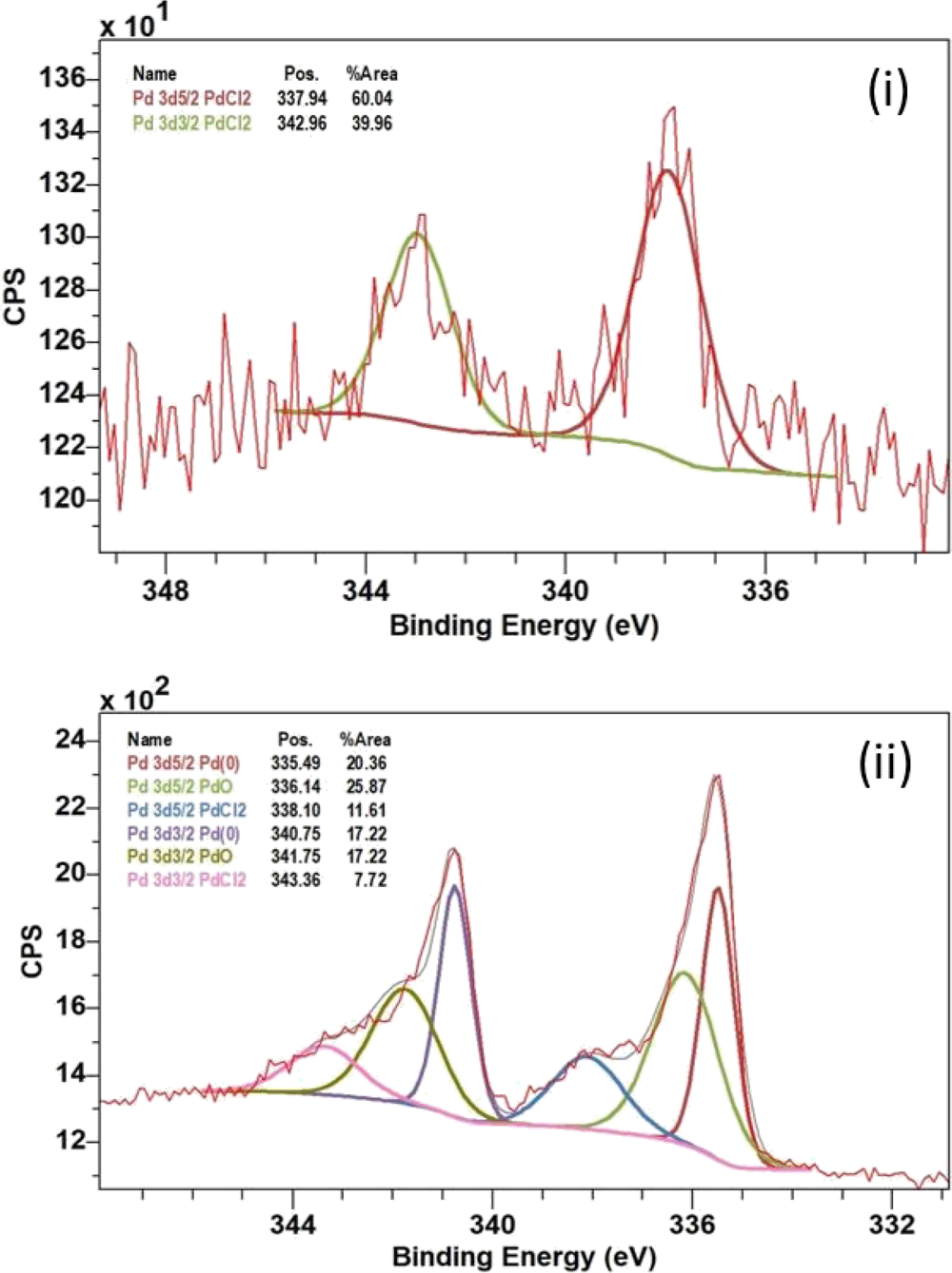
XPS spectra (Pd 3d_5/2_ and Pd 3d_3/2)_ of (i) +0.6 V vs SCE modified CB NPs and (ii) −0.1 V vs SCE modified CB NPs where the peaks with a binding energy of 335.49 eV (Pd 3d_5/2_) and 340.75 eV (Pd 3d_3/2_) indicate Pd^0^.

The samples in addition to unmodified CB NPs were characterized using ESEM/EDX and ICP-MS with results shown in Table 1. The ESEM/EDX analysis indicated that no palladium was detected in the unmodified sample, as expected as it had not been in contact with the PdCl_2_ solution. The sample held at −0.1 V exhibited the highest average weight % ratio Pd/C of 0.09 ± 0.009 followed by the sample held at +0.6 V with an average weight % ratio Pd/C of 0.03 ± 0.005. Analysis by ICP-MS showed a similar trend with the measured Pd concentration of 0.01, 2.75, and 17.67 ppm for the samples of unmodified CBNPs, held at 0.6 V vs SCE during chronoamperometry and at −0.1 V vs SCE, respectively. The Pd detected in the sample held at +0.6 V reflects the residual trace adsorbed palladium(II) left after the washing procedure. This level of Pd(II) appears to remain regardless of the extent of washing, suggesting adsorption to the CB surface, possibly via oxygen moieties. To further analyze the samples and determine the oxidation state of the Pd detected, XPS analysis was conducted on +0.6 and −0.1 V modified samples, shown in Figure 5. The data suggest that trace palladium was detected on the unmodified sample similar to results determined by the ESEM/EDX and ICP-MS analysis.

**Table 1:**
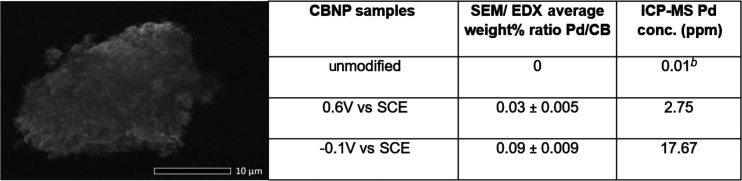
SEM Image of Agglomerated CBNPs Obtained from a 24 h Chronoamperometric Scan Conducted at −0.1 V (vs SCE) in a Solution of 0.5 mM PdCl_2_, 0.01 M KCl, and 0.01 M HCl^a^

a The table displays palladium content determined by SEM/ EDX and ICP-MS analysis of the unmodified CBNPs, the sample held at +0.6 V vs SCE during chronoamperometry, and the sample held at −0.1 V vs SCE.

b Measurement equivalent to zero within error.

The Pd 3d XPS characterization of the sample held at −0.1 V vs SCE indicated the presence of both Pd^0^ and Pd^2+^, with the latter associated with both PdCl_2_ and PdO. The peak with a lower binding energy (Pd 3d_5/2_) of 335.49 eV is assigned to the metallic palladium (Pd^0^) content while the peaks with a binding energy of 336.14 and 338.10 eV indicate the presence of PdO and PdCl_2_ respectively.^143−150^ The presence of Pd^0^ suggests that during impact events the potential was sufficiently negative to facilitate the reduction of Pd^2+^ to Pd^0^, which may have later (partially) oxidized to form PdO. Analysis of the sample held at +0.6 V vs SCE demonstrated the presence of Pd^2+^ as PdCl_2_ (with binding energies 337.94 and 343.96 eV) but no Pd^0^. This supports the earlier conclusion from the ESEM/EDX and ICP-MS data that some residual PdCl_2_ persisted after washing and no palladium deposition had occurred during collision at +0.6 V vs SCE.

### 3.3 Synthesis and Testing of Pd/CB NP Catalysts

**Figure 6:**
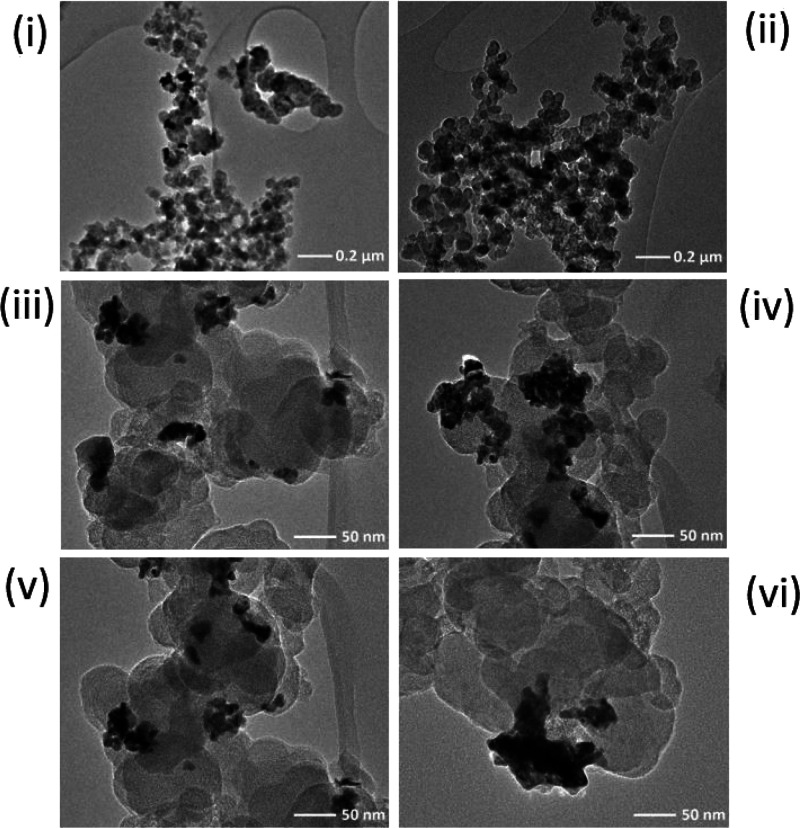
Typical TEM images (i–vi) of the 168 h palladium-modified carbon nanoparticles where the darker regions represent deposited Pd metal.

To sufficiently scale-up the quantity of Pd/CB NPs fabricated for testing in the hydrogen evolution and Suzuki coupling reactions, a batch of Pd/CB NPs was produced from a longer-term, 24 h, and 168 h deposition experiment (see the Experimental Section). Subsequent analysis by TGA (see Supporting Information Section C Figure S2) suggested that the Pd/CB NPs contained 14% by mass of Pd. The TEM images (Figure 6i–vi) further highlighted the presence of palladium metal on some particles evident in the contrasting darker regions seen on the carbon core, confirmed with EDX mapping (see Supporting Information Section D Figure S3). Deposition did not occur consistently across all Pd/CB NPs as some CB NPs were void of Pd suggesting that not all CBNPs collided with the GC electrode during the investigation or alternatively some collisions resulted in little to no Pd deposition. Figure 6 (TEM images of the impacted particles) suggests that the Pd deposits formed were not singular nanoparticles evenly distributed across the surface of the CB particles, but rather deposits covering sections of the impacting particle. Using the tilt function of the TEM, it was observed that these growths followed the curvature of the carbon particle rather than directly protruding out from the particle surface (Figure 6vi), and future work will explore further details of the Pd deposition to shed light on the mechanism of metal deposition during impact.

As a simple test of the catalytic capabilities of the Pd/CB NPs as catalysts without further treatment, samples were used for both hydrogen evolution and Suzuki coupling reactions. For the HER, 24 h modified Pd/CB NPs were compared with commercial 10 wt % Pd on CB catalyst. The test was conducted in a solution of 0.01 M H_2_SO_4_ and 0.09 M K_2_SO_4_ and showed the characteristic proton reduction profile for both the commercial standard and the impact synthesized sample (see Supporting Information Section E).

**Figure 7:**
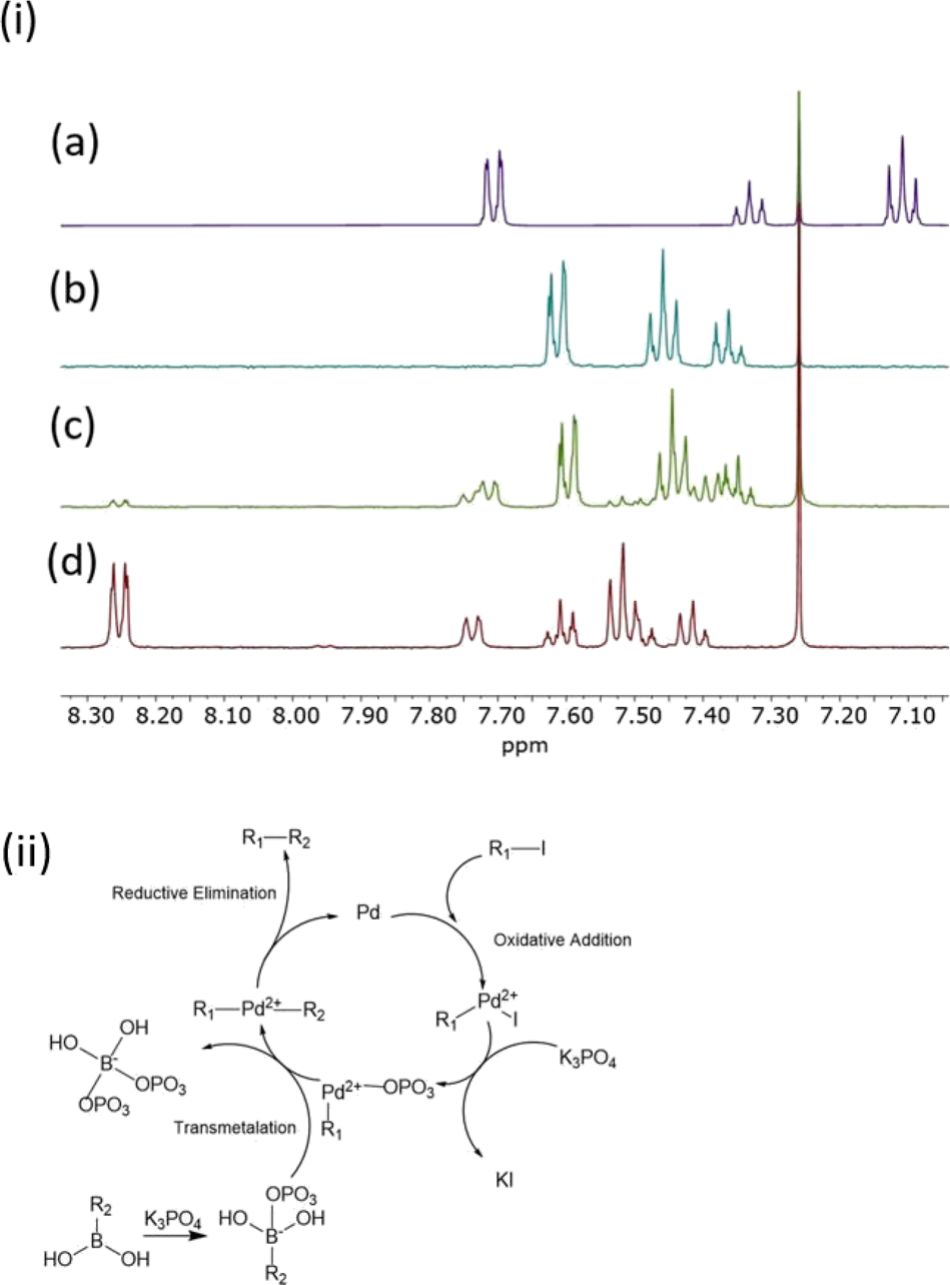
(i) ^1^H NMR spectrum of (a) iodobenzene, (b) biphenyl product taken from doi:10.13018/BMSE000506,^151,152^ (c) product extracted during the Suzuki reaction using Pd/CB NPs, and (d) displaying the ^1^H NMR analysis of the benzeneboronic acid reactant used in the investigation. The ^1^H NMR was performed using CDCl_3_ identified by the singlet at 7.26 ppm and (ii) schematic of the Suzuki reaction mechanism.

For the Suzuki reaction, 30 mg of 168 h modified Pd/CB NPs was used to catalyze the reaction between benzeneboronic acid (phenylboronic acid), iodobenzene, and potassium phosphate to produce biphenyl.^16,17,37^ This was achieved under 80 °C reflux where the resulting product was identified using ^1^H NMR. It should be noted that the quantity of Pd/CB NPs used is not optimized: at 14 wt % Pd this quantity of NPs provides *ca*. 4.2 mg of Pd, chosen to produce sufficient product for convenient handling and analysis.^37^ It was determined that the Pd/CB NPs had successfully catalyzed the reaction producing a mixture of biphenyl and residual benzeneboronic acid, evidenced by ^1^H NMR spectra of the product mixture (Figure 7i, spectrum (c)) which indicated that the iodobenzene had reacted and was no longer present in the final product as the signals at 7.10 ppm (spectrum (a)) are no longer observed. The multiplets at 7.60, 7.45, and 7.35 ppm in both the reference spectrum (b) and the product spectrum (c) suggest the presence of biphenyl. The additional signals can be attributed to the excess benzeneboronic acid initially used in the reaction.

### 3.4 Palladium Recovery from Low Concentration of PdCl_2_

Finally, the use of the impact method as a means to recover Pd from solution was studied. To examine the change in PdCl_2_ concentration during the impact deposition process, and hence Pd recovery from solution, 26 h chronoamperometric scans were conducted with and without CBNPs where a flow rate of nitrogen at 5 L min^–1^ (selected due to the range of flow rate gauges available) was maintained to ensure the same level of agitation. A 500 mL solution containing 0.01 M potassium chloride, 0.01 M hydrochloric acid, and 0.4 mM palladium(II) chloride (determined by ICP-OES) was used. The lower initial concentration determined by ICP-OES may be due to the presence of high levels of potassium ions in the samples causing a suppression of the Pd signal.^153,154^ At regular intervals throughout the experiments, 3 mL aliquots were extracted and filtered with 0.45 μm syringe filters to prepare samples for ICP-OES analysis.

**Figure 8:**
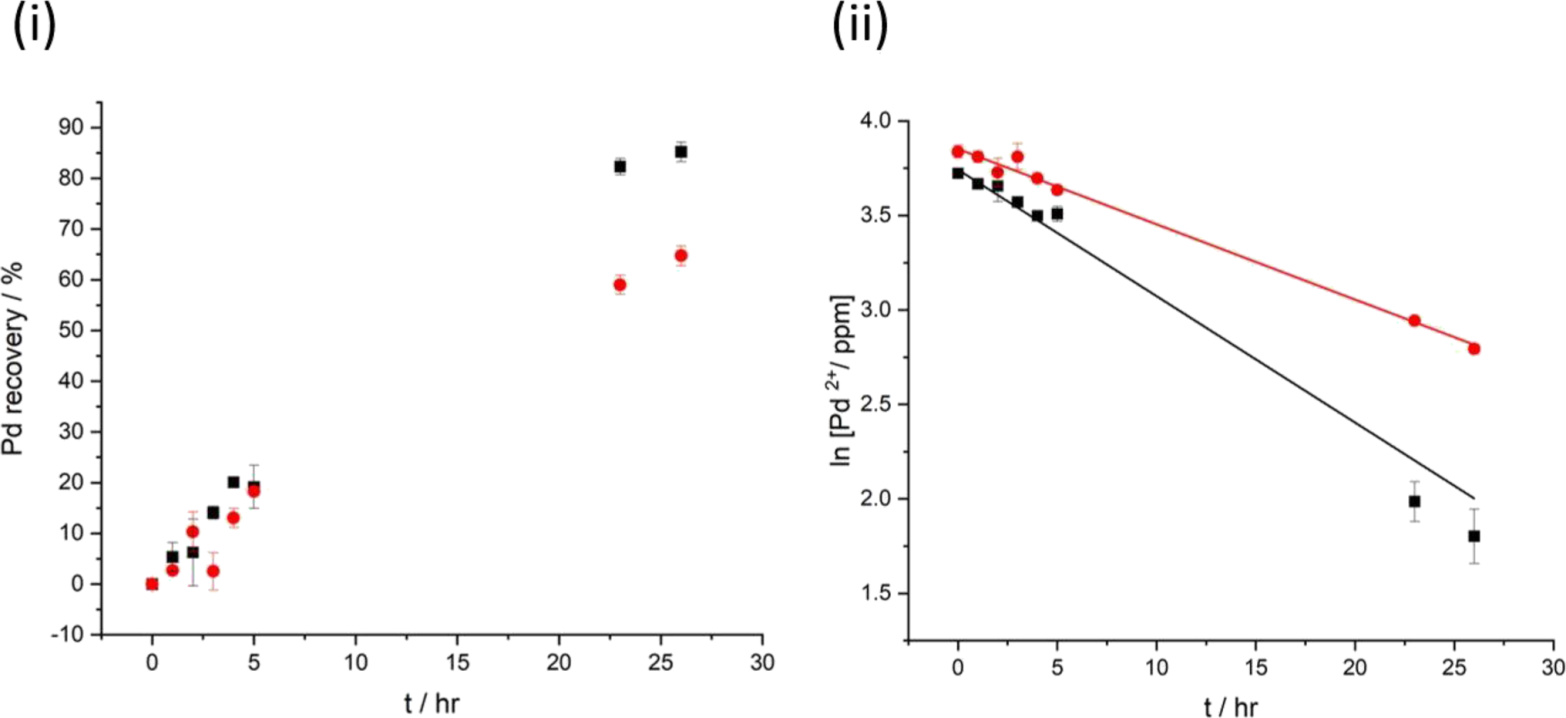
(i) Percentage of Pd recovered from PdCl_2_ during a 26 h chronoamperometric scan conducted with (black square ■) and without (red circle ●) CB NP particles. (ii) ln [Pd^2+^] vs time based on the data in (i) where the best fit lines from linear regression are given by ln[Pd^2+^/ppm] = 3.74–0.067 *t* for (black square ■) and ln[Pd^2+^/ppm] = 3.85–0.04 *t* for (red circle ●).

Figure 8i shows the percentage of Pd^2+^ recovered during a 26 h chronoamperometric scan with and without the addition of CB NPs. Figure 8ii shows the depletion in Pd^2+^ concentration in logarithmic form: the NP-mediated experiment showing an enhancement in the recovery rate of a factor of approximately 1.7, for these nonoptimized conditions. Despite each individual nanoimpact resulting in the deposition of *ca*. 10^5^ Pd atoms, the high number of impacts has a significant effect on the overall rate of removal of Pd from solution. Future work will seek to quantify the relationship between the experimental parameters and recovery rate.

The background deposition occurring on the graphite substrate electrode during chronoamperometric scans with and without CB NPs was studied via ESEM/EDX (see Supporting Information Section F). A comparison of Figure S6 displaying a polished graphite electrode with Figures S7 and S8 showing an electrode held in solution under potential for as long as 168 h confirms that Pd deposition occurs on the electrode resulting in a heavily modified surface. Figure S9 shows a side profile of both graphite electrodes (modified with and without CBNPs) indicating that the extent of Pd deposition on the background electrode is more prominent without the presence of particles. An average Pd thickness of 2.2 μm with larger growths (some exceeding 10 μm) was seen on the electrode without CBNPs in comparison to an average thickness of 0.5 μm seen on the electrode used with CB NPs present.

## 4 Conclusions

Initial deposition studies conducted on glassy carbon showed that bulk deposition of Pd occurred at potentials negative of *ca*. +0.4 V (vs SCE), in good agreement with the literature.^88,140,142^ Subsequent impact electrochemistry studies with CB NPs showed that transient impact events resulting in Pd deposition also commenced at *ca*. +0.40 V (vs SCE). Analysis of the resulting transient peaks determined an average charge of −14.8 ± 2.1 fC was observed at the “switch on/off potential” of +0.38 V (vs SCE) increasing to −49.0 ± 15.6 fC at a potential of −0.1 V (vs SCE).

Direct evidence of impact-mediated Pd deposition on the CB NPs was obtained via ESEM, EDX, TEM, ICP-MS, and XPS analysis of the Pd/CB NP sample. The impact experiment was successfully scaled up to produce enough Pd/CB NPs to test as a catalyst through large-volume long-term chronoamperometry, and synthesized Pd/CB NPs were characterized using TEM and TGA confirming a metal loading of 14 wt % Pd. The particles were then used to demonstrate direct catalytic application via the hydrogen evolution and Suzuki coupling reactions.

Finally, Pd recovery via nanoimpacts was investigated. Although not optimized to recover the maximum amount of Pd from solution, it was found that comparing Pd recovery with and without CB NPs over a 26-h period, the NP-mediated method increased recovery from *ca.* 65% to *ca.* 85%. This demonstrates the potential for single-entity electrochemistry to be a useful recovery method for metals, and future work will investigate its optimization and develop semiempirical equations for practical use as well as exploring applications for the deposited metal such as using 3D structured substrate electrodes that could be used for energy storage (e.g., recovered Ni, Mn, and Co for battery materials).

## Supporting Information Available

Transient current signal analysis using 9 and 33 μm CF; reductive peak charge and coverage analysis; TGA of the 168 h modified Pd/CB NP sample; TEM/EDX of impacted CB NPs; LSV of HER using modified CB NPs; cleaning CV conducted before HER investigation; and ESEM/EDX of the electrode used in Pd recovery investigation (PDF)

## Abbreviations

PGM: platinum group metals
CB NPs: carbon black nanoparticles
SCE: saturated calomel electrode
CV: cyclic voltammetry
LSV: linear sweep voltammetry
GC: glassy carbon
CF: carbon fiber
ESEM: environmental scanning electron microscopy
EDX: energy-dispersive X-ray spectroscopy
TEM: transmission electron microscopy
XPS: X-ray photoelectron spectroscopy
ICP-MS: inductively coupled plasma mass spectrometry
ICP-OES: inductively coupled plasma optical emission spectrometry
