# Supplementary material for: Electrochemical Metal Recycling: Recovery of Palladium from Solution and In Situ Fabrication of Palladium-Carbon Catalysts via Impact Electrochemistry
Source: J Am Chem Soc. 2022 Sep 30;144(40):18562–74. doi: 10.1021/jacs.2c08239 (PMC9562286; doi:10.1021/jacs.2c08239)
Supplement: Supplementary file 1 — ja2c08239_si_001.pdf [file ja2c08239_si_001.pdf]

Electrochemical metal recycling: recovery of palladium from solution and in situ fabrication of palladium-carbon catalysts via impact electrochemistry

Abiola V. Oladeji, James M. Courtney, Marcos Fernandez-Villamarin, Neil V. Rees*

School of Chemical Engineering, University of Birmingham, Edgbaston, Birmingham B15 2TT. United Kingdom

SUPPLEMENTARY INFORMATION

* Corresponding author

Email: n.rees@bham.ac.uk

Tel: +44 (0)121 4145325

Section A: Analysis of transient current signals from impacting CBNPs on 9 µm and 33 µm CF electrodes

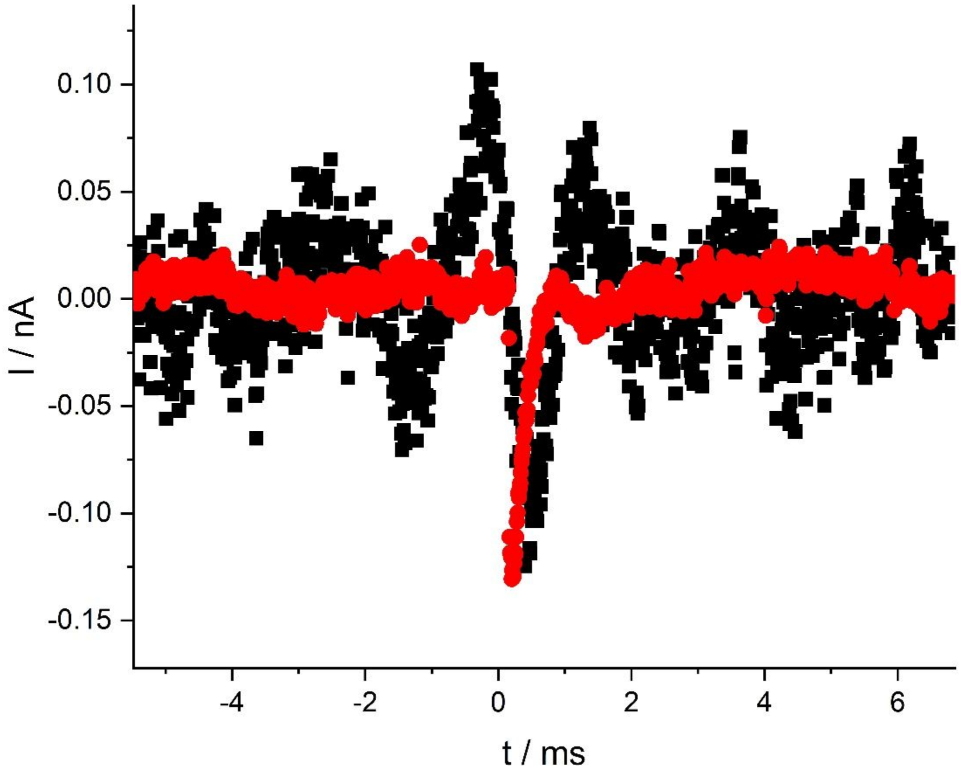

Figure S1. Illustrative unfiltered peaks conducted on a 9 µm (•) and 33 µm (▪) carbon fibre electrodes during a 30 s chronoamperometric scan held at -0.1 V vs. SCE. The peaks have been time and background current corrected to show the similarity in peak heights occurring on a 9 µm and 33 µm CF electrode. All scans were conducted in a solution containing 0.5 mM $PdCl_2$, 0.01 M KCl and 0.01 M HCl.

Impact studies were conducted on both 9 µm and 33 µm carbon fibre (CF) electrodes where Figure S1 displays examples of the isolated unfiltered peaks. The use of a larger microelectrode resulted in a higher background current thus decreasing the signal to noise ratio, however due to the larger area a higher number of transient events could be observed. Analysis of the peaks conducted with 50 pM of 50 nm CB NPs on the 33 µm and 9 µm CF electrodes using the unfiltered data indicated individual peaks were virtually identical in height, shown in figure S1 where a typical example of an isolated unfiltered peak conducted on a 9 µm CF and 33 µm CF where an average peak current of (-0.13 ± 0.03) nA and (-0.11 ± 0.07) nA were determined respectively.

Section B: Peak charge and coverage calculation of impacting CBNPs

Calculation used to determine the palladium coverage during nanoparticle impact:[1]

$$S = 4\pi R^2 \quad (1)$$

$$Q = \int I dt = ezN \quad (2)$$

$$N_{mono} = 0.7405 \frac{S}{\pi r^2} \quad (3)$$

$$\theta = \frac{Q}{5.924}\left(\frac{r}{R}\right)^2 \quad (4)$$

where S is the nanoparticle surface area, R the NP radius, Q the associated reductive charge, t the spike duration, e the electronic charge, z = 2 the number of exchanged electrons per reduced palladium atom,[2,3] θ the coverage, r = 2.10 Å the palladium atomic radius,[4] N the number of reduced Pd atoms and N_{mono} the number of palladium atoms in a monolayer based on a 74.05 % fcc surface coverage .[2] The average coverage was calculated for impacts at different potentials.

Parameter / unit	CB NP (-0.1 V vs. SCE)
S / m^2	7.85×10^{-15}
Q / C	-4.02×10^{-14}
N	1.26×10^{5}
N_{mono}	4.20×10^{4}
θ / %	300

Table T1. Example calculation of palladium coverage on the surface of CB NPs. The calculated values for the NP surface area, reductive peak charge, number of Pd deposited, number of Pd in monolayer and coverage equivalent are shown. This calculation was conducted using unfiltered raw peak data.

Section C: TGA analysis of 168 hr modified samples

CB NPs modified during the 168 hr chronoamperometry scan conducted at -0.1 V vs. SCE were characterised using thermogravimetric (TGA) analysis.

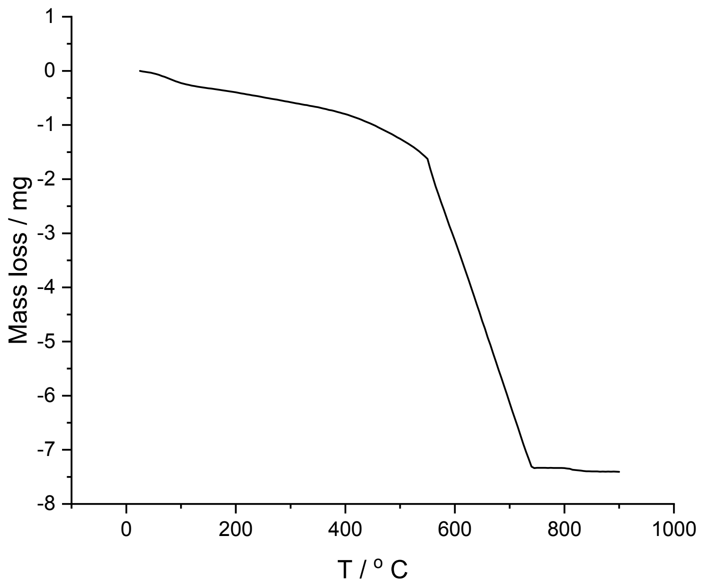

Figure S2. TGA thermogram of the 168 hr palladium modified carbon nanoparticles. From this, a 13.9 % palladium coverage was determined based on a mass change from 8.6 mg to 1.2 mg upon reaching 900 °C.

Section D: EDX analysis performed during TEM of Pd/CB NPs

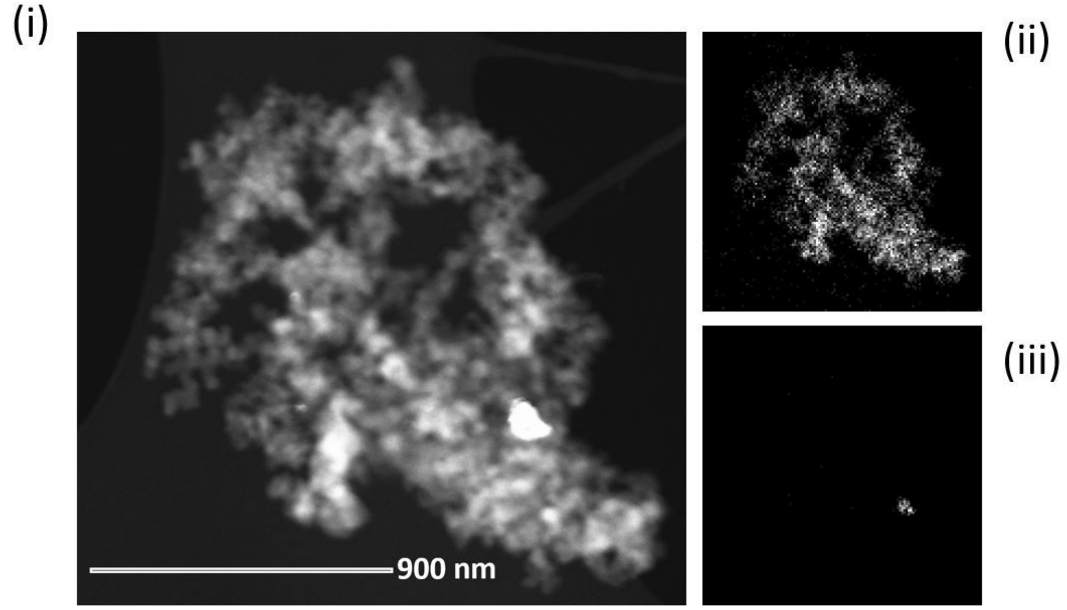

Figure S3 (i) TEM showing an agglomerated Pd/CB NPs where the CBNPs (ii) and Pd deposition (iii) have been identified using EDX mapping.

Section E: Hydrogen evolution reaction (HER) catalysis investigation

Pd/CB NPs were investigated as possible electrocatalysts for the hydrogen evolution reaction (HER) by dropcasting Pd/CB NPs onto a 3 mm GC working electrode and performing cyclic voltammetry in a solution of 0.1 M H_2SO_4 and 0.09 M K_2SO_4 in a three-electrode cell with a SCE reference and a graphite rod counter electrode. Figure S4 shows the resulting voltammograms recorded using GC electrodes which were modified with CB NPs, Pd/CB NPs, and commercial 10 % Pd/CB catalyst. Initial Electrochemical cleaning scans (100 cycles) were performed at a scan rate of 500 mV s^{-1} between 0.8 V to -0.27 V vs. SCE before conducting CVs in order to remove surface contaminants (See Figure S5).

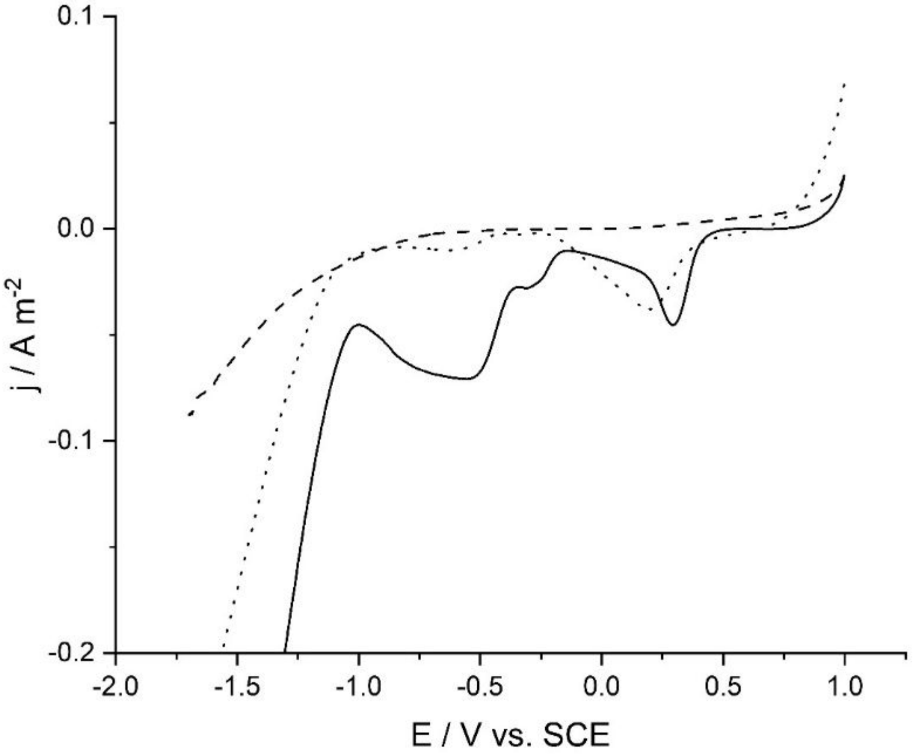

Figure S4. Reductive cyclic voltammetry scans conducted between 1.0 V to -1.7 V vs. SCE at a scan rate of 100 mV s^{-1} on the surface of unmodified CBNPs/GC (----), -0.1 V (vs. SCE) modified CBNPs/ GC (—)and commercial 10 % Pd modified CBNPs/ GC (····). This was conducted in a solution of 0.01 M H_2SO_4 and 0.09 M K_2SO_4 after an initial 100 cycle cleaning scan conducted at 500 mV s^{-1}.

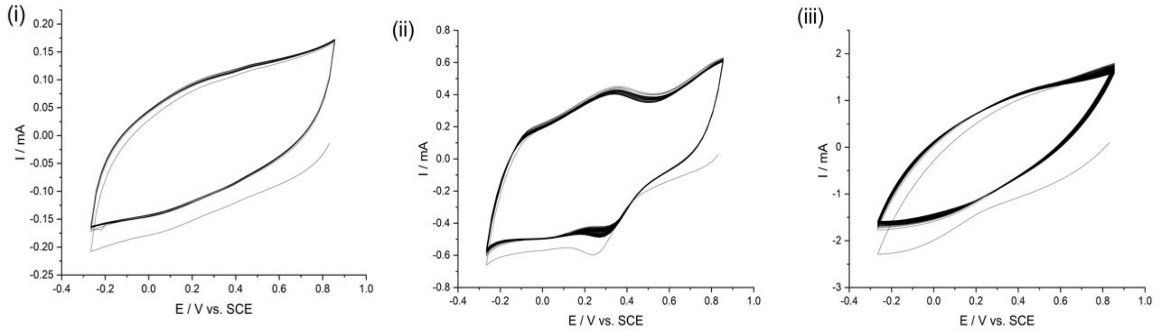

Figure S5. The 100 cycle CV cleaning scans conducted for (i) unmodified CBNPs/ GC, (ii) -0.1 V vs. SCE modified GC and (iii) commercial 10% Pd modified CBNPs/ GC. A scan rate of 500 mV s^{-1} was used between the potential window 0.8 V to -0.27 V vs. SCE in a solution of 0.1 M sulfuric acid and 0.09 M potassium sulfate.

Figure S4 shows that a higher overpotential is required for the reduction of protons on the surface of the unmodified CB NPs with onset at ca. -1.0 V (vs. SCE). However, for both the Pd/CB NPs and commercial 10 % Pd/CB NPs a more positive initial peak at ca. 0.4 V (vs. SCE) associated with the reduction of oxide layers can be identified. A feature at ca. -0.2 V (vs. SCE) associated with hydrogen adsorption,[5-7] and the similarity of the CV response for both samples further suggests that the impact voltammetry technique was sufficiently able to modify the CB NPs at -0.1 V vs. SCE and significantly that these modified particles show electrocatalytic activity. The onset of hydrogen evolution on the Pd modified surfaces can be observed at ca. -0.3 V followed by additional HER occurring on the GC surface at -0.1 V vs. SCE.[8]

Section F: ESEM/ EDX analysis of graphite substrate electrode

This section provides the study conducted on the deposition of palladium on substrate electrodes used during impact electrochemistry investigations. ESEM/ EDX was used to analyse a polished graphite plate substrate electrode and electrodes after use in the 168 hr chronoamperometry investigations (both with and without nanoparticles).

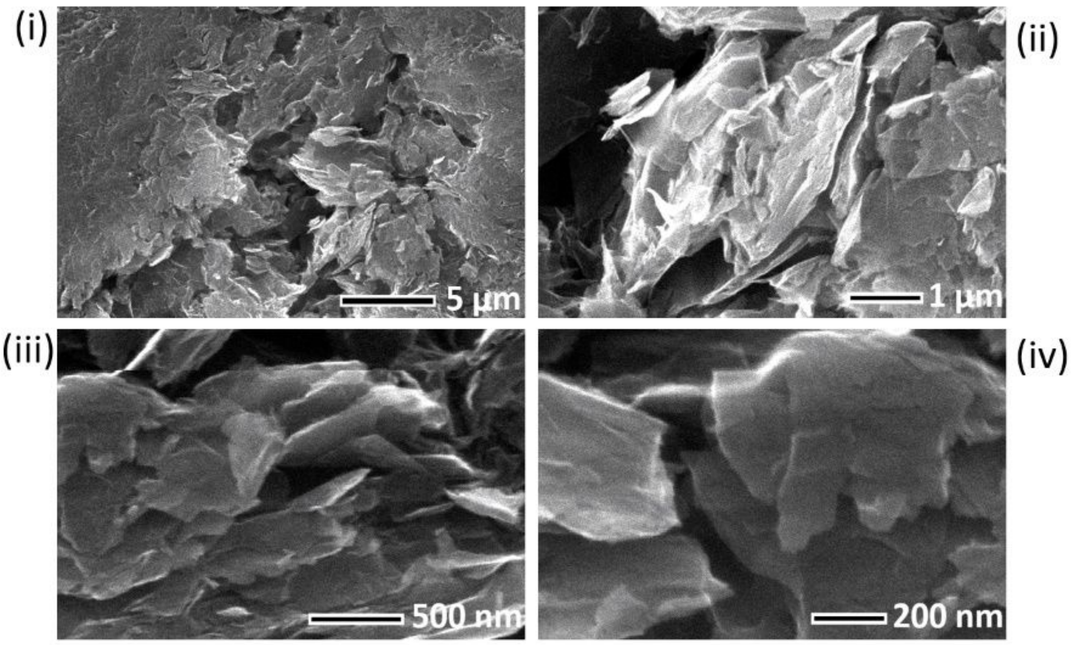

Figure S6. ESEM images of a polished graphite electrode at magnification factors of (i) 5×10^3, (ii) 2×10^4, (iii) 5×10^4, and (iv) 10^5. This electrode was polished using a diamond paste 3 µm and alumina suspensions of 1 µm, 0.3 µm and 0.05 µm sequentially, on a micro-cloth pad.

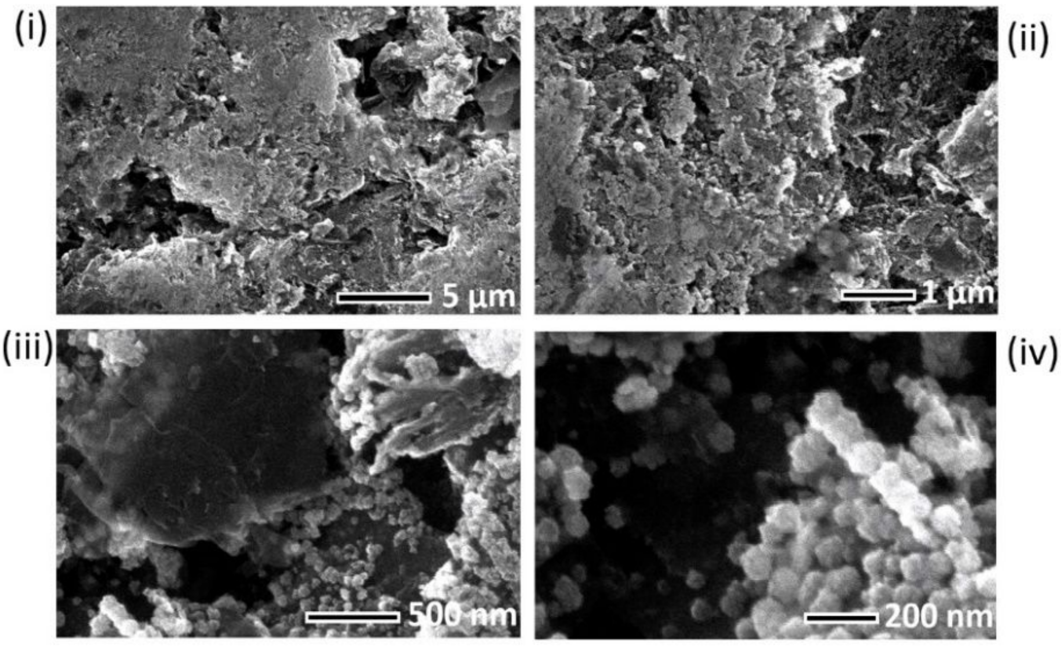

Figure S7. ESEM images of a modified graphite electrode after use in a 168 hr chronoamperometry scan at magnification factors of (i) 5×10^3, (ii) 2×10^4, (iii) 5×10^4, and (iv) 10^5. The chronoamperometry scan was conducted at -0.1 V vs. SCE in a palladium plating solution in the presence of 20 nM CB NPs.

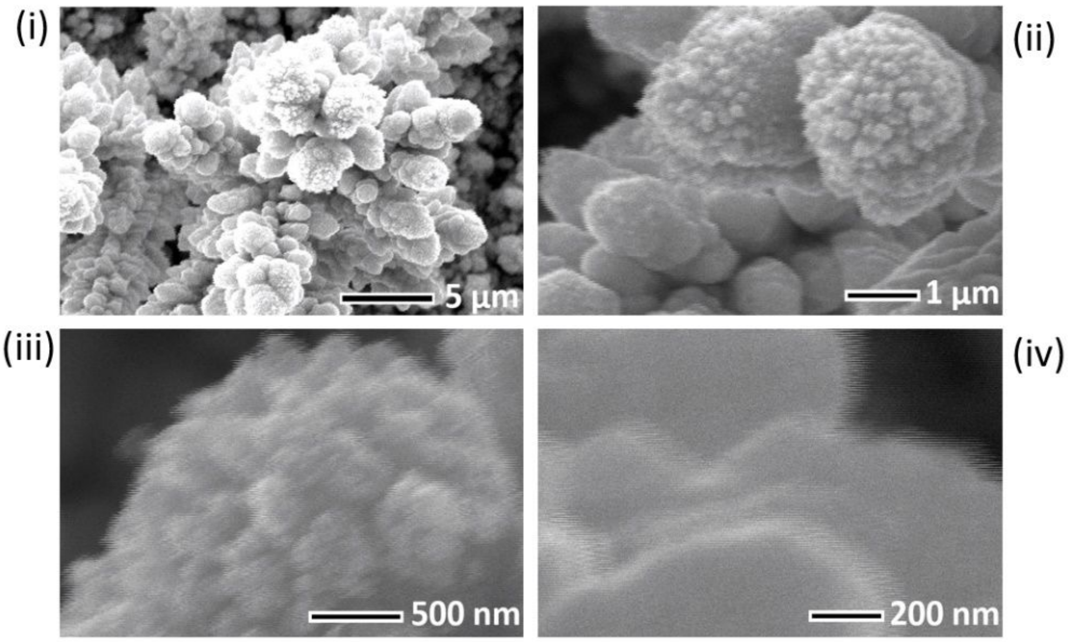

Figure S8. displays ESEM analysis of a modified graphite electrode after use in a 168 hr chronoamperometry scan at magnification factors of (i) 5 × 10^3, (ii) 2 × 10^4, (iii) 5 × 10^4, and (iv) 10^5. The chronoamperometry scan was conducted at -0.1 V vs. SCE in a palladium plating solution without CB NPs.

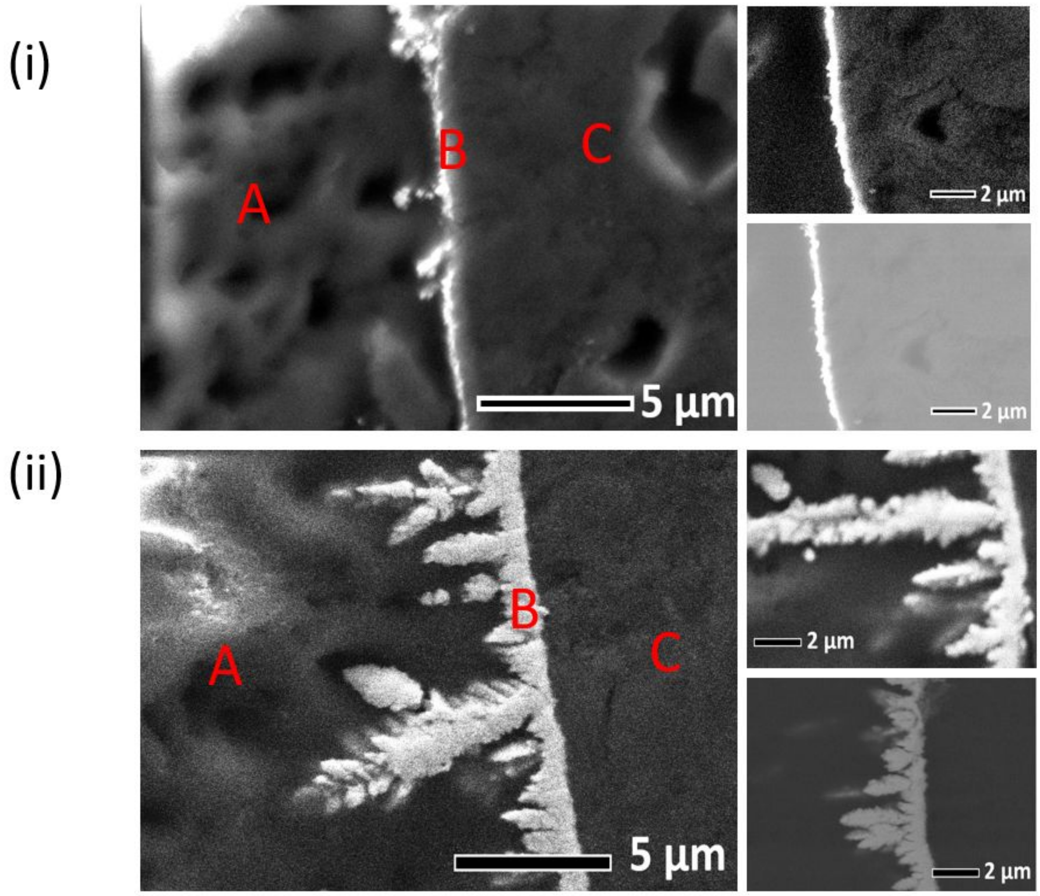

Figure S9. displays SEM/EDX analysis of a modified graphite electrode after use in a 168 hr chronoamperometry with (i) and without (ii) the presence of 20 nM CBNPs where A, B and C represent the epoxy, Pd deposition and graphite region respectively where EDX was performed to determine the Pd content. A lower Pd weight% of 27% was detected on the electrode surface where CB NPs were used in comparison to deposition occurring without particles where a 99.6% Pd weight% was detected. The epoxy regions of both samples had much lower Pd content of 0% (-0.4%) for sample (i) and 11.6% for sample (ii) and region C (graphite) indicated 0.19 % and 1.39 % in sample (i) and (ii) respectively.

Section G: Speciation of Palladium (II)

In the reaction solution containing 0.5 mM of $PdCl_2$ and 0.02 M of HCl, the most likely palladium species are: $[Pd(H_20)_6]^{2+}$, $[Pd(H_20)_5Cl]^{+}$, $[Pd(H_20)_4Cl_2]$, $[Pd(H_20)_3Cl_3]^{-}$, and $[Pd(H_20)_2Cl_4]^{2-}$. The approximate equilibrium concentrations of the species can be calculated via their respective stability constants,[9,10] given the mass balance requirement that the total Pd(II) and Cl^- concentrations must add up to 0.5 mM and 0.021 M respectively.

	$\log_{10}\beta_n$	Species	Calculated Equilibrium Concentration / mol dm^{-3}
$[Pd(H_20)_6]^{2+} + Cl^- = [Pd(H_20)_5Cl]^+ + H_2O$	4.47	$[Pd(H_20)_5Cl]^+$	1.57×10^{-6}
$[Pd(H_20)_6]^{2+} + 2Cl^- = [Pd(H_20)_4Cl_2] + 2H_2O$	7.74	$[Pd(H_20)_4Cl_2]$	5.68×10^{-5}
$[Pd(H_20)_6]^{2+} + 3Cl^- = [Pd(H_20)_3Cl_3]^- + 3H_2O$	10.20	$[Pd(H_20)_3Cl_3]^-$	3.19×10^{-4}
$[Pd(H_20)_6]^{2+} + 4Cl^- = [Pd(H_20)_2Cl_4]^{2-} + 4H_2O$	11.50	$[Pd(H_20)_2Cl_4]^{2-}$	1.23×10^{-4}
		$[Pd(H_20)_6]^{2+}$	2.74×10^{-9}
		Cl^-	0.0194

Table T2. Complexation equilibria, associated stability constants and calculated equilibrium concentrations of Pd-chloro complexes in the reaction solution.

References

1. A. V. Oladeji, J. M. Courtney and N. V. Rees, *Electrochim. Acta*, 2022, **405**, 139838.
2. S. Thiagarajan, R. Yang and S. Chen, *Bioelectrochemistry*, 2009, **75**, 163-169.
3. T. Wang, A. Chutia, D. J. L. Brett, P. R. Shearing, G. He, G. Chai and I. P. Parkin, *Energy Environ. Sci.,* 2021, **14**, 2639-2669.
4. *CRC Handbook of Chemistry and Physics*, ed. J. R. Rumble, CRC Press, Boca Raton, 102 edn., 2021.
5. S. S. Kumar, S. U. B. Ramakrishna, B. R. Devi and V. Himabindu, *Ionics*, 2018, **24**, 3113-3121.
6. S. Sarkar and S. C. Peter, *Inorg. Chem. Front.*, 2018, **5**, 2060-2080.
7. K. N. Mahesh, R. Balaji and K. S. Dhathathreyan, *Int. J. Hydrogen Energy*, 2016, **41**, 46-51.

8. T. Alemu, B. D. Assresahegn and T. R. Soreta, *Port. Electrochim. Acta*, 2014, **32**, 21-33.
9. M. Wojnicki, K. Paclawski, E. Rudnik, K. Fitzner, *Hydrometallurgy*, 2011, **110**, 56–61.
10. S. Gu, X.-P. Wang, Y.-Z. Wei, B.-Z. Fang, *Sci. China Chem.,* 2014, **57**, 755-762
